# Speech Signal and Facial Image Processing for Obstructive Sleep Apnea Assessment

**DOI:** 10.1155/2015/489761

**Published:** 2015-11-17

**Authors:** Fernando Espinoza-Cuadros, Rubén Fernández-Pozo, Doroteo T. Toledano, José D. Alcázar-Ramírez, Eduardo López-Gonzalo, Luis A. Hernández-Gómez

**Affiliations:** ^1^GAPS Signal Processing Applications Group, Universidad Politécnica de Madrid, 28040 Madrid, Spain; ^2^ATVS Biometric Recognition Group, Universidad Autónoma de Madrid, Madrid, Spain; ^3^Respiratory Department, Sleep Unit, Hospital Quirón, Málaga, Spain

## Abstract

Obstructive sleep apnea (OSA) is a common sleep disorder characterized by recurring breathing pauses during sleep caused by a blockage of the upper airway (UA). OSA is generally diagnosed through a costly procedure requiring an overnight stay of the patient at the hospital. This has led to proposing less costly procedures based on the analysis of patients' facial images and voice recordings to help in OSA detection and severity assessment. In this paper we investigate the use of both image and speech processing to estimate the apnea-hypopnea index, AHI (which describes the severity of the condition), over a population of 285 male Spanish subjects suspected to suffer from OSA and referred to a Sleep Disorders Unit. Photographs and voice recordings were collected in a supervised but not highly controlled way trying to test a scenario close to an OSA assessment application running on a mobile device (i.e., smartphones or tablets). Spectral information in speech utterances is modeled by a state-of-the-art low-dimensional acoustic representation, called i-vector. A set of local craniofacial features related to OSA are extracted from images after detecting facial landmarks using Active Appearance Models (AAMs). Support vector regression (SVR) is applied on facial features and i-vectors to estimate the AHI.

## 1. Introduction

Sleep disorders are receiving increased attention as a cause of daytime sleepiness, impaired work, and traffic accidents and are associated with hypertension, heart failure, arrhythmia, and diabetes. Among sleep disorders, obstructive sleep apnea (OSA) is the most frequent one [1]. OSA is characterized by recurring episodes of breathing pauses during sleep, greater than 10 seconds at a time, caused by a blockage of the upper airway (UA) at the level of the pharynx due to anatomic and functional abnormalities of the upper airway.

The gold standard for sleep apnea diagnosis is the polysomnography (PSG) test [2]. This test requires an overnight stay of the patient at the sleep unit within a hospital to monitor breathing patterns, heart rhythm, and limb movements. As a result of this test, the Apnea-Hypopnea Index (AHI) is computed as the average number of apnea and hypopnea episodes (partial and total breath cessation episodes, resp.) per hour of sleep. This index is used to describe the severity of patients' condition: low AHI (AHI < 10) indicates a healthy subject or mild OSA patient (10 ≤ AHI ≤ 30), while AHI above 30 is associated with severe OSA. However, polysomnography monitoring is costly and invasive and removes the patients from their normal sleeping environment; therefore, faster, noninvasive, and less costly alternatives have been proposed for early OSA detection and severity assessment. In this work we explore alternative procedures for estimating the AHI using voice and facial data. These procedures are studied for an OSA-symptomatic population (i.e., individuals that have been referred to a sleep unit for PSG); therefore, our ultimate goal will be to help in setting priorities to proceed to the PSG diagnosis based on the expected OSA severity (i.e., stratification). This will ensure a better treatment of patients according to their needs and will be particularly relevant in some countries as Spain where waiting lists for PSG may exceed one year [3].

Although central obesity and an excess of regional adipose tissue are considered major risk factors for OSA, craniofacial abnormalities and an altered UA structure are also recognized as important interacting factors in OSA pathogenesis. The rationale of using image and speech analysis in OSA assessment can be found on works such as Lee et al. [4, 5] and Davidson et al. [6], where the evolutionary changes in physiological characteristics such as craniofacial morphology or acquisition of speech are connected to the appearance of OSA from an anatomical basis.

In [7] authors applied sophisticated volumetric analysis on magnetic resonance imaging (MRI) of the upper airway soft tissue structures in control and sleep apnea subjects. By means of statistical tests they reported physiological differences between the groups for tongue volume (*P* < 0.001), lateral pharyngeal walls (*P* < 0.001), and total soft tissue (*P* < 0.001). Lowe et al. [8] assessed the interaction between craniofacial structures by computed tomography of lateral cephalometry, tongue, soft palate, and upper airway size in control subjects and sleep apnea patients, finding differences with respect to tongue, soft palate, and upper airway volumes. In [9], authors studied oropharyngeal soft tissues profile by means of cephalometric analysis in order to detect differences between control and sleep apnea individuals. Significant differences were related to the length of the soft palate (*P* < 0.001), area of the soft palate (*P* < 0.001), and distance of close contact between the tongue and soft palate (*P* < 0.001). These previous works rely on advanced imaging techniques, allowing a detailed examination of bony and soft tissue structures. However, these procedures are generally cost-expensive, time-consuming, and invasive for patients due to radiation exposure. As a simple alternative, based on the correlation between craniofacial anthropometry and photogrammetry, it can be reasonable to explore the use of image processing as noninvasive, faster, and more readily accessible techniques. Face characterization technologies have already tested on the diagnosis of genetic syndromes [10]. In the reference work Lee et al. [4] compare the craniofacial morphological phenotype of sleep apnea and control populations applying photogrammetry on frontal and profile digital photographs of the head of subjects under study. After manually landmarking images, they computed a total of 71 craniofacial measurements representing the dimension and relationship of craniofacial regions including face, mandible, maxilla, eyes, nose, head, and neck. They reported correlation to OSA severity (AHI) specially for some measures as neck depth (*r* = 0.51, *P* < 0.001) or neck perimeter (*r* = 0.50, *P* < 0.001). Later on, Lee et al. [5] selected the most discriminative features among these 71 measurements extended with another 62 craniofacial measurements also by photogrammetry. Using logistic regression they reported 76.1% correct classification between OSA and non-OSA.

The particular facial phenotype in OSA individuals has also been found to correlate with specific upper airway structures using magnetic resonance imaging (MRI), for example, a correlation between tongue volume and midface width [11]. These physical alterations as craniofacial abnormalities—dental occlusion, longer distance between the hyoid bone and the mandibular plane, relaxed pharyngeal soft tissues, large tongue base, and so forth—generally cause a longer and more collapsible upper airway (UA). Consequently, abnormal or particular speech features in OSA speakers may be expected from the altered structure or function of their UA.

Early approaches to speech-based OSA detection can be found in [12, 13]. In [12] authors used perceptive speech descriptors (related to articulation, phonation, and resonance) to correctly identify 96.3% of normal (healthy) subjects, though only 63.0% of sleep apnea speakers were detected. The use of acoustic analysis of speech for OSA detection was first presented in [14, 15]. Fiz et al. [14] examined the harmonic structure of vowels spectra, finding a narrower frequency range for OSA speakers, which may point at differences in laryngeal behavior between OSA and non-OSA speakers. Later on Robb et al. [15] presented an acoustic analysis of vocal tract formant frequencies and bandwidths, thus focusing on the supralaryngeal level, where OSA-related alterations should have larger impact according to the pathogenesis of the disorder.

These early contributions have driven recent proposals for using automatic speech processing techniques in OSA detection such as [16–21]. Different approaches, generally using similar techniques as in speaker recognition [22], have been studied for Hebrew [16, 21] and Spanish [17] languages. Results have been reported for different types of speech (i.e., sustained and/or continuous speech) [16, 18, 20], different speech features [16, 19, 20], and modeling different linguistic units [18]. Also speech recorded from two distinct positions, upright or seated and supine or stretched, has been considered [20, 23].

In this paper we explore the use of both voice and facial features for OSA assessment. Considering the capability of mobile devices (tablets, smartphones, smartwatches, etc.) for an easy collection of physiological data, such as voice and face images, these techniques could be useful for very simple and noninvasive preliminary assessment of OSA. Frontal and profile images and voice recordings were collected for a large population of 285 male Spanish speakers suspected to suffer from OSA and derived to a Sleep Disorders Unit. Pictures and recordings were collected in a supervised but not highly controlled scenario to resemble a mobile device scenario.

Deciding which features can be useful to estimate the AHI represents a different challenge for facial and vocal characteristics. Automatic facial characterization to estimate the AHI can rely on a set of facial features where previous research (Lee et al. [4, 5]) has already linked to the craniofacial phenotype of sleep apnea. However, existing research has not been able to clearly identify a set of specific acoustic features for OSA speakers. To tackle this difficulty, in our research we analyze a corpus of four speech sentences that was specifically designed to include a set of characteristic sounds in OSA voices. These four sentences were designed following the reference research in [12, 13], where Fox et al. identify a set of possible speech descriptors in OSA related to articulation, phonation, and resonance. So, for example, the third sentence in our corpus includes mostly nasal sounds to detected resonance anomalies (more details on the design criteria for this corpus can be found in [19]). Once focused on an OSA-specific acoustic space, state-of-the-art speaker's voice characterization technologies, previously tested and demonstrated to be effective in the estimation of other speaker's characteristics such as height [24] and age [25], were used to estimate the Apnea-Hypopnea Index (AHI). We can support this approach by considering that similar methods have been demonstrated to outperform other approaches (such as the use of formant analysis or GMMs models) for detecting other physiological variables as age, height, or BMI.

Besides facial and voice features we also evaluated AHI prediction using the available clinical variables: age, height, weight, BMI (Body Mass Index), and neck circumference. This allows us to compare AHI estimation when using only facial features, speech features, or clinical variables and also when combining all the available information. To our knowledge this is the first time that AHI prediction is explored by analyzing both speech signal and facial image processing techniques and considering their combination with other clinical indicators of sleep apnea.

## 2. Methods

### 2.1. Subjects and Experimental Design

The population under study is composed of 285 male subjects referred to a pneumonologist and presenting symptoms of OSA such as excessive daytime sleepiness, snoring, choking during sleep, or somnolent driving. Clinical variables (age, height, weight, BMI, and cervical perimeter) were collected for each individual. This database has been recorded in Hospital Quirón Málaga (Spain) since 2010. All the work was performed strictly following the ethical consideration of the center and the participants were notified about the research and their agreement obtained. Statistics of the clinical variables used in this study are summarized in Table 1.

The diagnosis for each patient was confirmed by specialized medical staff through polysomnography (PSG), obtaining the AHI on the basis of the number of apnea and hypopnea episodes. Two types of data were collected from the patients, as explained in the following:

- Acoustic data: patients' speech was recorded prior to PSG. All speakers read the same 4 sentences and sustained a complete set of Spanish vowels [i, e, a, o, u]. As there is no clear set of specific acoustic features characterizing OSA speakers, these four speech sentences were designed following the reference research in [12, 13], where Fox et al. identify a set of possible speech descriptors in OSA speakers related to articulation, phonation, and resonance. So, for example, the third sentence in our corpus includes mostly nasal sounds to detected resonance anomalies (more details on the design criteria for this corpus can be found in [19]). Recordings were made in a room with low noise and patients at an upright or seated position. Recording equipment was a standard laptop with USB SP500 Plantronics headset. Speech was recorded at a sampling frequency of 50 kHz and encoded in 16 bits. Afterwards it was downsampled to 16 kHz before processing.
- Photographic data: frontal and profile digital photographs of the head were obtained before the speech recordings, also at the same normal hospital room without any particular illumination condition. Differently from [4, 5], no special actions were taken beyond a simple control for patients' front and profile photographs and some instructions to guarantee that the neck area is visible in the profile image. No calibration action for allowing the conversion from pixel measurements to metric dimensions (e.g., measuring the distance from the camera) was taken, and manual identification (by palpation) of facial landmarks was also avoided. A standard Logitech QuickCam Pro 5000 webcam was used to collect images with a size of 640 × 480 pixels and a color depth of 24 bits.

Data has been collected since 2010 and several operators (up to six) have been involved, always blinded to the results of the polysomnography. This can guarantee that our results are not dependent on a particular acquisition process. However, we have not had the opportunity of testing the same subject several times.

### 2.2. Problem Formulation 

We are given a training dataset of acoustic/facial features and Apnea-Hypopnea Index (AHI) information *S*_tr_ = {**x**_*n*_, *y*_*n*_}_*n*=1_^*N*^, where **x**_*n*_ ∈ *ℜ* ^*p*^ denotes the feature vector representation (acoustic/facial) of the training dataset and *y*_*n*_ ∈ *ℜ* denotes the corresponding value of AHI.

The goal is to design an estimator function *f* for AHI, such that, for an acoustic/facial feature vector from an unseen testing subject **x**_tst_, the difference between the estimated value of a Apnea-Hypopnea Index $\hat{y}=f(x_{tst})$ and its truth or actual value *y* is minimized.

### 2.3. Acoustic Features

Speaker recognition technologies usually represent the acoustic information in a speech utterance as a sequence of feature vectors corresponding to the short-term spectral envelope of the embedded sounds. In this study Mel-Frequency Cepstrum Coefficients (MFCC) extended with their first order derivative will be used, as they are commonly adopted in most of the automatic speaker recognition systems [26, 27].

Moreover, as different utterances naturally exhibit sequences of MFCC feature vectors with different lengths, they are generally transformed into fixed-length vectors **x** representing all the relevant acoustic information in the utterance (all the variability and therefore the vector space of this representation are called total variability [28]). This will also be very convenient in our case since it allows a fixed-length acoustic vector **x** to be used as input to the estimator function *f*, which makes estimation much simpler.

**Figure 1:**
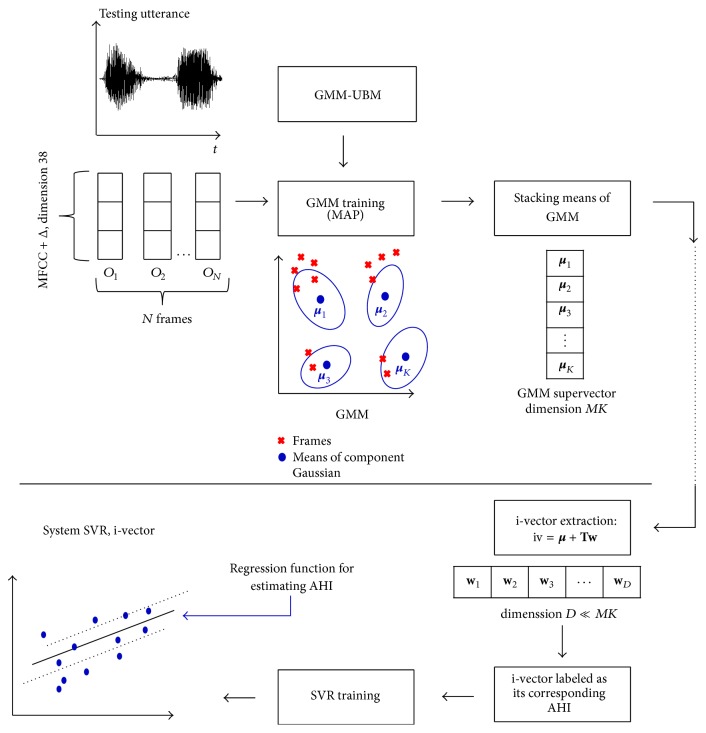
Acoustic representation of utterances and SVR training.

The most common approach for this transformation, called i-vectors, was followed in our study, and it is depicted in Figure 1. I-vectors were developed on the success of modeling the probability density function of sequences of feature vectors as a weighted sum of Gaussian component densities, Gaussian Mixture Models (GMM). As illustrated in Figure 1, a GMM representing an utterance from a particular speaker can be obtained through adaptation of a universal background model (GMM-UBM) trained on a large speaker population [29]. Once a GMM is adapted from a GMM-UBM using the utterances of a given speaker, a supervector will be just the stacked pile of all means of the adapted GMM [26]. As the typical number of Gaussian components in a GMM for speaker recognition is between 512 and 2048, and dimension of MFCC acoustic vector takes values from 20 to 60, speech utterances will then be represented by high-dimensional vectors **x** of sizes 10 K to 120 K.

Beyond high-dimensional supervectors, a new paradigm called i-vector has been successfully and widely used by the speaker recognition community [28]. It relies on the definition of a low-dimensional total variability subspace **T** and can be described in the GMM mean supervector space by

(1) $$\begin{array}{c} m=\mu +Tw, \end{array}$$

where **m** is the GMM supervector for an utterance, ***μ*** is the utterance-, speaker-, health-, and clinical condition-independent supervector obtained from a universal background model GMM-UBM. **T** is a rectangular matrix of low rank, which defines total variability space (representing all sources of variability in speech recordings). The matrix **T** is estimated using the EM algorithm in a large training dataset. An efficient procedure for training **T** and MAP adaptation of i-vectors can be found in [22]. **w** is a random vector having a standard normal distribution *N*(0, *I*), which are composed of total factors. These total factors represent the speaker's voice characteristics. The total factors are defined by their posterior distribution conditioned to Baum-Welch statistic for a given utterance. The mean of this distribution corresponds to i-vectors.

Compared to supervectors, the total variability modeling using i-vectors has the advantage of projecting the high dimensionality of GMM supervectors into a low-dimensional subspace, where most of the speaker-specific variability is captured.

Both supervectors and i-vectors have been successfully applied to speaker recognition [28], language recognition [30], speaker age estimation [25], speaker height estimation [24], and accent recognition [31]. Consequently, we believe that the success of using i-vectors in challenging tasks, where speech contains significant sources of interfering intraspeaker variability (speaker weight, height, etc.), is a reasonable guarantee for exploring its use in estimating the Apnea-Hypopnea Index (AHI). Furthermore, as the same microphone was used for all recording and all the speakers read the same corpus of four sentences, both channel and phonetic variabilities are minimized so it is reasonable to think that i-vectors will capture characteristics from sounds that can be more affected by OSA. The interested reader may find additional discussion on this topic in [32] together with a comparison when using supervectors and i-vectors to predict AHI and other clinical variables.

### 2.4. Speech Databases for Development

To guarantee that all the relevant speaker information is contained in the low-dimensional vector space, the development of i-vectors requires training the total variability subspace **T** using recordings from a large speaker population database representative of a wide range of different speakers, channels, or noisy conditions. However, in our clinical environment, the acoustic space only includes continuous/read Spanish speech recorded with a single microphone and in a relatively noise-free environment. Therefore, our variability subspace should mainly represent the rich articulation variability of a variety of Spanish sounds pronounced by a wide range of speakers. So far, for development, we used different databases containing microphonic speech sampled at 16 KHz, covering a wide range of phonetic variability from continuous/read Spanish speech (see, e.g., ALBAYZIN [33], as it was one the databases we used). The development dataset used to train the total variability subspace and UBM includes 25451 speech recordings from 940 speakers. Among them 126 speakers, certainly diagnosed with OSA and not used for tests, were also included so that the **T** matrix also reflects the variability due to the presence or absence of OSA-specific characteristics of speech.

### 2.5. Facial Features

One of the most important stages in automatic face recognition is feature extraction. In our case, the objective is to have a specific compact and structured representation of craniofacial characteristics able to describe both inter- and intraclass variability for OSA and non-OSA individuals. There are three main types of facial features in state-of-the-art automatic face recognition [34]: holistic features, local features, and features derived by statistical models. In this study we have taken as reference the work from Lee et al. in [4, 5], so local features are used. However, as described below, our major differences compared to the research in [4, 5] are the use of supervised automatic image processing and the definition of more robust craniofacial measurements adapted to our less controlled photography capture process.

**Figure 2:**
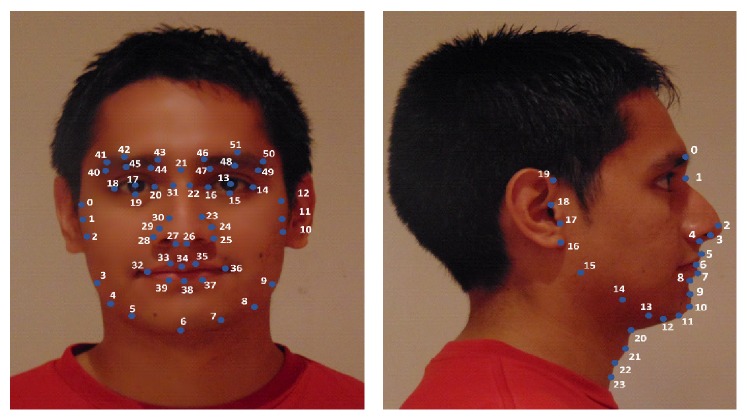
Landmarks on frontal and profile view.

A first critical step for extracting local facial features is to identify a set of relevant landmarks on images of subjects under study. The database of facial images contains frontal and profile digital photographs of 285 male subjects, that is, 570 digital photographs needed to be processed to obtain landmarks. Manual annotation of all images, as done in [4, 5], can be tedious and, even if done by skilled personal, it is prone to errors due to subjectivity. Consequently, we decided to use a widely used automatic landmarking method, first introduced by Cootes et al. in 2001 [35], based on Active Appearance Model (AAM) [36]. Based on a priori knowledge of landmark positions, AAM combines a statistical model, which represents the variation of shape and texture of the face (object), with a gradient-descent fitting algorithm. As Figure 2 shows, in AAMs for frontal and profile photographs we use a grid of 52 landmarks taken from a general face identification system and a set of 24 landmarks including specific marks for the neck area, respectively.

During the training stage, frontal and profile AAMs were built from a set of manually annotated photographs using the aam_tools software [37]. During the fitting stage, starting from a reasonable landmark initialization, the AAM algorithm iteratively corrects the appearance parameters by minimizing the squared error to represent the texture of the target face. Although the AAM performs well for representing shape and appearance variations of an object, the model is location-sensitive to face's position. In this study this effect is increased because photographs were not taken following a highly controlled procedure (illumination conditions, control of distance from the camera, and control of frontal and profile position). Hence a human-supervised stage was found necessary in order to supervise and, if necessary, correct some large deviations in the automatically generated landmarks.

Once landmarks were generated we proceed to extract a set of local features based on previous studies [4, 5] but, as stated before, adapted to our less controlled photographic process. More specifically, a main difference with Lee et al. research is that in [4] photography was performed using a professional camera and following a highly controlled procedure: frontal position was achieved controlling that subject's facial landmark nasion was aligned along the subject alignment plane while ensuring both ears were seen equally from the front, and a laser pointer head-clip pointing to calibrated markings on the side wall was used to ensure the profile views were perpendicular to the frontal views. This highly controlled scenario contrasts to our objective of exploring the possibility of using speech signals and images captured in a more informal way using standard portable devices. Besides that, there are two another remarkable differences compared to [4]. Firstly, we did not include any calibration procedure that could have allowed us to convert pixel measurements to metric dimensions. This could have been done as in [4] by including a procedure to measure the distance from the camera or by sticking a calibration marker in the patient's face (as a circular nylon washer). We decided not to follow any of these calibration processes trying to explore results in a more usable scenario, but we are aware that this makes calibrated measurements unavailable, which according to [5] should have provided better results. A second decision that we also made looking for a better user experience was to avoid any manual identification (by palpation) of facial landmarks.

Consequently, based on the results in [4, 5] and considering our limited photography capture process, only uncalibrated measures (i.e., relative measurements or angles) were used, and three alternative craniofacial measurements related to those identified by Lee et al. were designed. These measurements are described in the following.

#### 2.5.1. Cervicomental Contour Area

**Figure 3:**
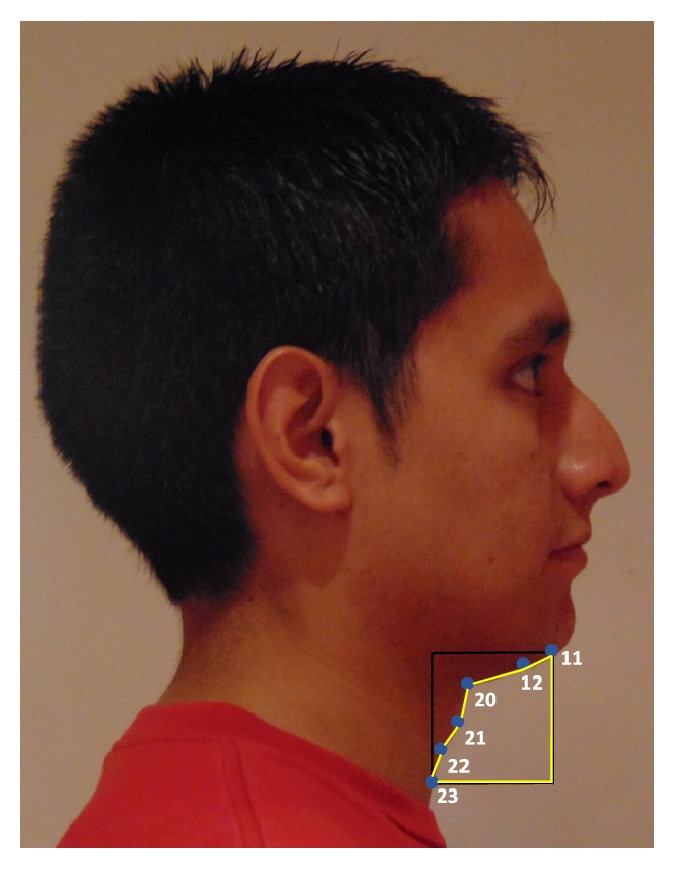
Cervicomental contour area.

One of the anatomical risk factors for OSA is the fat deposition on the anterior neck [38]. In [4, 5] this risk factor is captured by cervicomental angle (neck-cervical point-mentum), where an increase of neck fat deposition causes an increase of this angle. However, considering our limited photography capture process, it is extremely difficult to detect points such as: cervical point, thyroid, cricoid, neck plane, or sternal notch involved in the cervicomental region. Consequently, we defined an alternative measurement, more robust to both our image capture and automatic landmarking processes. This measure was defined using a contour in the cervicomental region traced by six points, placed equidistantly, which were annotated with high reliability following our semiautomatic AAM method (see Figure 3). In this cervicomental measure, the area of a rectangle defined by bottom left point 23 and upper right point 11 is used to normalize the area defined by points 11 12 20 to 23 and the right and low sides of the 23–11 rectangle. This results in an uncalibrated measurement with a value that decreases as the fat deposition on the anterior neck increases.

#### 2.5.2. Face Width

**Figure 4:**
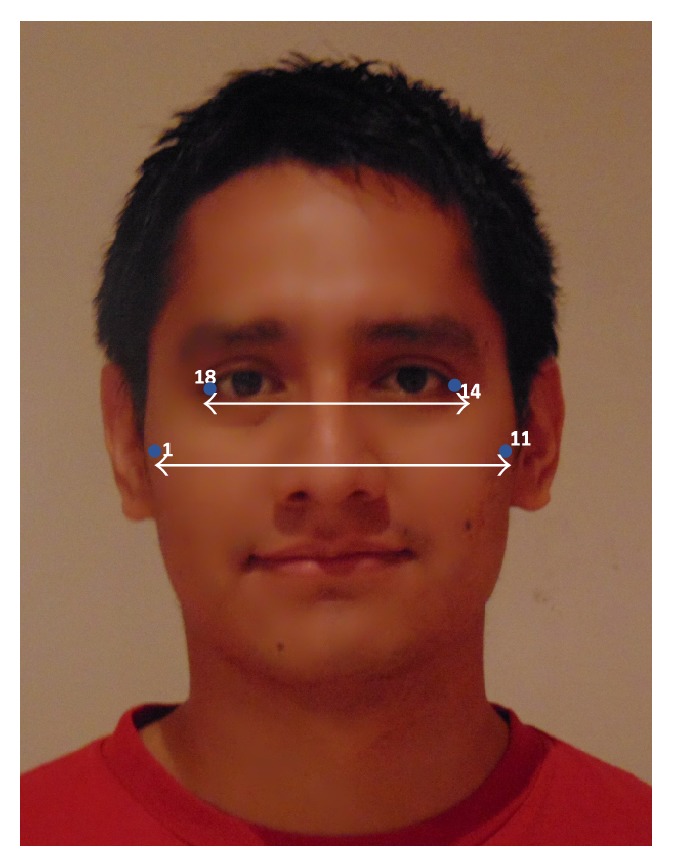
Face width.

In Lee et al. [11], magnetic resonance imaging (MRI) was used to study the relationship between surface facial dimensions and upper airway structure in subjects with OSA. Significant positive correlations were detected between surface facial dimensions and upper airway structures, in particular midface width and interocular width. Based on Lee's work we used these two facial dimensions to define a* face width* uncalibrated measurement as the midface width to interocular width ratio. The corresponding landmarks and measures are depicted in Figure 4.

#### 2.5.3. Tragion-Ramus-Stomion Angle

**Figure 5:**
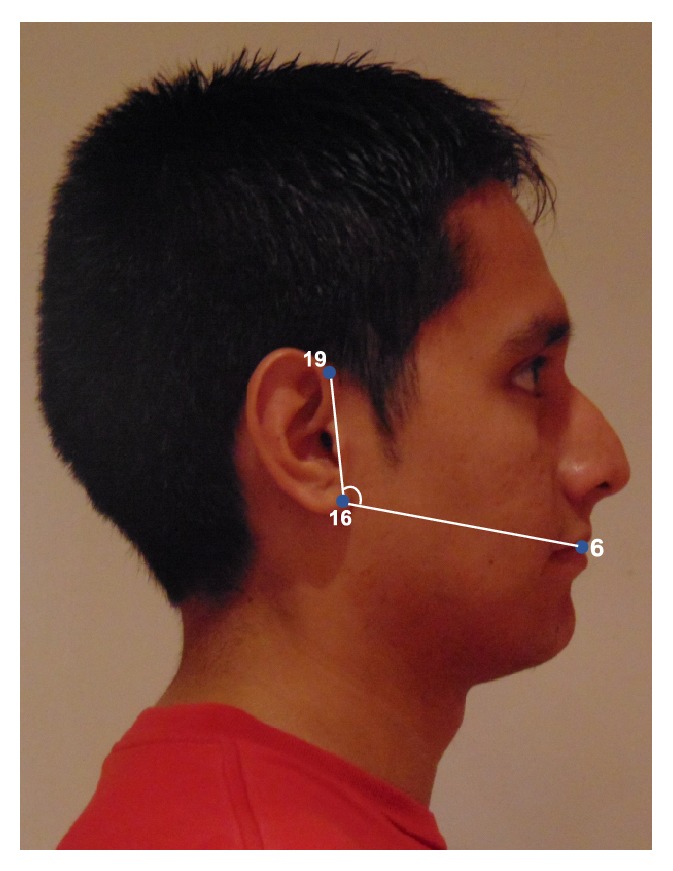
Tragion-ramus-stomion angle.

In Lowe et al. [8], it was reported that patients with OSA had retracted mandibles, which is related to the inclination of the occlusal plane and the ANB angle (measuring the relative position of the maxilla to mandible). Based on Lowe's work we proposed an uncalibrated measure (i.e., an angle) intended to capture, to some extent, the characteristic mandible position or mandibular retraction in OSA individuals. To define this angle we selected a set of landmarks that not only are related to the posterior displacement of the mandible but also could be accurately detected by our automatic landmarking process on the photographs without need of prior marking. The proposed measurement (Figure 5) is the angle between the line* ramus*-*stomion* (points 16 and 6) and the* ramus*-*tragion* (points 16 and 19).

#### 2.5.4. Facial Feature Vector

**Figure 6:**
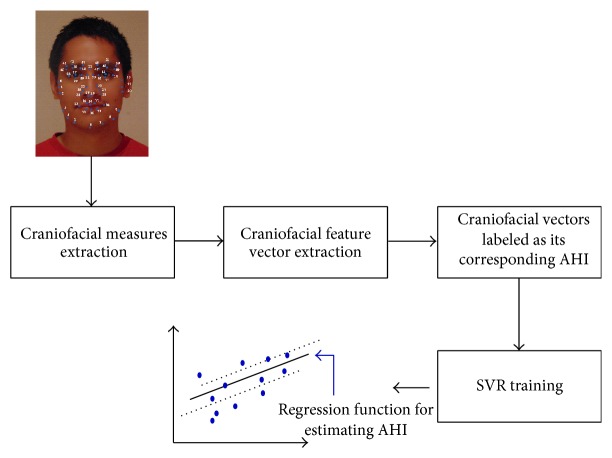
Craniofacial AHI prediction model.

The facial feature vector then consists in the combination of the 3 craniofacial measurements described before. This feature vector is the input to a SVR regression model used to predict the AHI. The craniofacial features extraction process is illustrated in Figure 6.

### 2.6. Regression Using SVR

For estimating the Apnea-Hypopnea Index (AHI) in our OSA database we followed a similar approach as the one used in [24] and [25] for height and age, respectively. As depicted in Figures 1 and 6, once facial/acoustic features are represented by their corresponding feature vector, **x**, support vector regression (SVR) is employed as the estimator function *f* ^*j*^ for the Apnea-Hypopnea Index *y* ^*j*^.

Support vector regression (SVR) is a function approximation approach developed as a regression version of the widely known Super Vector Machine (SVM) classifier. Firstly, the input variable is mapped onto a high-dimensional feature space by using a nonlinear mapping. The mapping onto a high-dimensional space is performed by the kernel function. The kernel yields the new high-dimensional feature by a similarity measure between the points from the original feature space. Once the mapping onto a high-dimensional space is done then a linear model is constructed in this feature space by finding the optimal hyperplane in which most of the training samples lie within *ϵ*-margin (*ϵ*-insensitive zone) around this hyperplane [39]. The generalization of SVR's performance depends on a good setting of two hyperparameters, *ϵ* and *C*, and the kernel parameters. The parameter *ϵ* controls the width of the *ϵ*-insensitive zone, used to fit the training data. The width of the *ϵ*-insensitive zone determines the level of accuracy of approximation function. It relies entirely on the target values of the training set. The parameter *C* determines the trade-off between the model complexity, controlled by *ϵ*, and the degree to which deviations larger than the *ϵ*-insensitive zone are tolerated in the optimization of the hyperplane. Finally, the kernel parameters depend on the type similarity measure used.

In this paper, support vector regression (SVR) is applied to estimate the Apnea-Hypopnea Index (AHI) and linear kernel is used to approximate the estimator function *f* ^*j*^.

The training and testing was implemented using LIBSVM [40] and the optimization of the hyperparameters of the SVR and the parameter of the RBF was performed by a grid search, which is a simply exhaustive searching through the subset of hyperparameters and parameters guided by a 5-fold cross-validation as performance metric.

### 2.7. Performance Metrics

The performance of the proposed prediction scheme was evaluated using both the Mean Absolute Error (MAE) of the subject's estimated AHI and the Pearson linear correlation coefficient (CC) between the actual and estimated values of AHI.

#### 2.7.1. Leave-One-Out Cross-Validation and Grid Search

**Figure 7:**
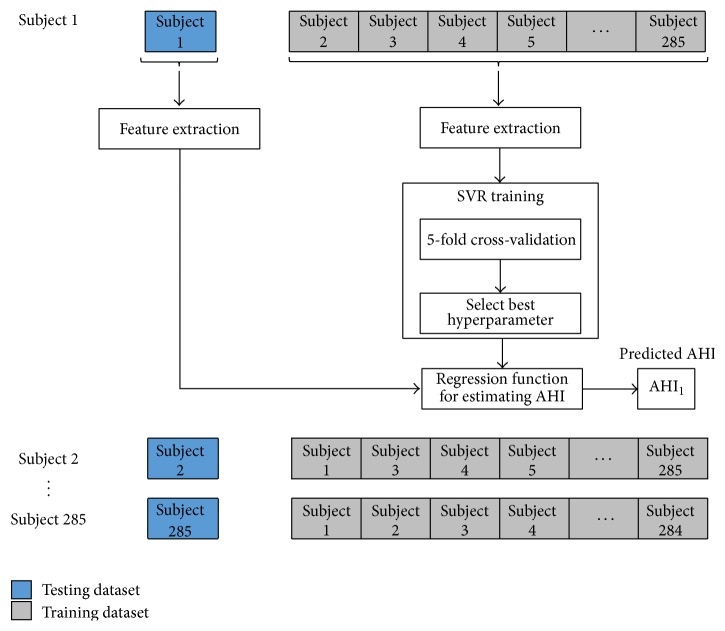
Representation of leave-one-out cross-validation and grid search process for training the regression model and predicting the AHI.

In order to validate the regression model we employed leave-one-out cross-validation. To do this, one subject is removed from dataset for testing data and the other for training data. Then, in order to find the optimum complexity of the model, we apply a 5-fold cross-validation on training data to find the optimal parameters values of the support vector regression model. For this purpose we implement a “grid search” on the hyperparameters of the SVR model using 5-fold cross-validation. The grid search consists of an exhaustive search through specified set of hyperparameters (*ϵ* and *C*) of the SVR model. Therefore, various pairs of hyperparameters values are tried and the one with the best cross-validation MAE is picked. After finding the optimal parameter value, we train the model using the optimal hyperparameter values and the training dataset. Finally, the testing dataset is used to predict the AHI assigned to each input by using SVR models trained solely from the training dataset. The whole process is repeated for all dataset and it is depicted in Figure 7.

## 3. Results

**Figure 8:**
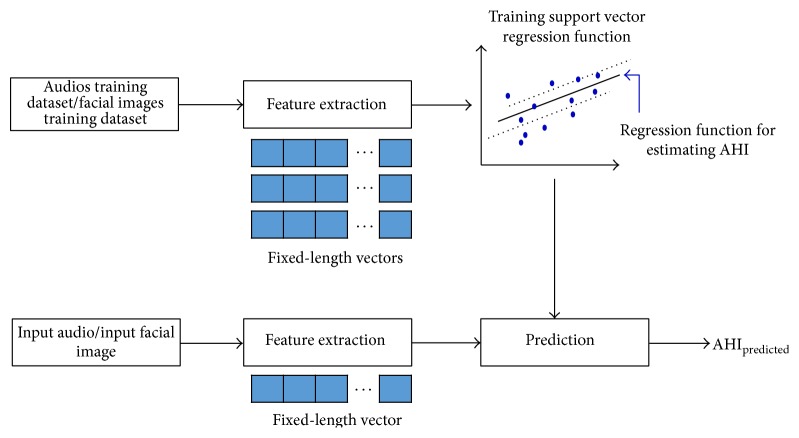
Description of training and testing phase in order to predict AHI.

During the testing phase, as it is shown in Figure 8, each subject is first processed by extracting its acoustic features/facial features and then the features extracted are stacked for obtaining fixed-length vectors. These vectors are used over the trained SVR models to predict the value of the subject's AHI.

Both to compare the system performance and to have a reference when predicting the AHI, similarly to Lee et al. work [5], we also trained SVR models using the available clinical variables as input vectors (age, Body Mass Index, and cervical perimeter), which are well known predictors for AHI. Mean Absolute Error (MAE) and Pearson correlation coefficient (CC) results when predicting AHI using these clinical variables are presented in Table 2.

Prediction results using i-vectors and craniofacial vectors for AHI prediction are listed in Tables 3 and 4.  Table 3 includes Mean Absolute Error and correlation coefficient results when using only the three uncalibrated craniofacial measurements (cervicomental contour area, face width, and tragion-ramus-stomion angle) and when combining these uncalibrated craniofacial measurements with the clinical variables (age, Body Mass Index, and cervical perimeter) in a single feature vector.

Results in Table 4 are given for speech acoustic representation using i-vectors with different dimensionality (from 400 to 30). Somewhat better results can be observed for low dimensionality (50) probably because of the limited number of speakers in the development databases. As in Table 4, performance results are also given when combining i-vectors and clinical variables. The analysis of these results shows a very weak prediction capability of OSA when using speech acoustic features.

## 4. Discussion

Our results indicate that facial features extracted from frontal and profile images can be better predictors of OSA than acoustic i-vectors features extracted from reading speech. Although different previous studies [20, 21], including ours [19], have reported good results using speech processing techniques for OSA assessment, our recent results, as those reported in this work using a large number of subjects recorded in a clinical practice scenario, only reveal a weak connection between OSA and speech. This fact has also been discussed in our research in [41] where only very weak correlations were detected between AHI and formant frequencies and bandwidths extracted from sustained vowels. Results reported in this paper, based on the more powerful acoustic representation of speech using i-vectors, seem to follow a similar trend. This has motivated us to address a critical review of previous research using speech processing for OSA assessment, presented in [41], where we report several limitations and methodological deficiencies that may have led to previous overoptimistic results. Considering now the features extracted from frontal and profile facial images, we can compare our results to those reported in [4] which, to the best of our knowledge, represent the first and only research reference in this field. From a functional point of view, our approach presents two major differences: (1) landmark identification is done using supervised automatic image processing instead of through precise manual identification as in [4], and (2) we use three uncalibrated photographic measurements, inspired by those selected by Lee et al. [5] but more suitable for a less controlled photography capture process. Besides these differences, overall, our results are close and follow a similar trend compared to those reported in [5]. As it can be seen in Tables 2 and 3, correlation coefficient (CC) and Mean Absolute Error (MAE) between truth and estimated AHI values are only slightly better when using only clinical variables (CC = 0.40; MAE = 12.32) than when only relying on uncalibrated facial measurements (CC = 0.37; MAE = 12.56). Furthermore, when clinical variables and uncalibrated measurements are combined together, a moderate increase in performance is observed (CC = 0.45; MAE = 11.97), though in [5] results are only given when combining calibrated measurements with clinical variables.

An important issue when contrasting our results is that we have focused on predicting the AHI (using SVR) while the objective in [5] is to classify or discriminate between subjects with OSA (AHI ≥ 10) and without OSA (defined by an AHI < 10) using logistic regression and classification and regression trees (CART). Therefore, we performed some additional tests using our estimated AHI value to classify subjects with OSA (truth AHI ≥ 10) and without OSA (truth AHI < 10). Classification results using this procedure are shown in Table 5. We are aware that better results should be obtained if a classification algorithm, as SVM, was used, instead of regression with SVR followed by classification using the estimated AHI, but we are only looking for some figures that allow us to draw some further comparisons.

Results in Table 5 show that this approach classified 70.8% of the subjects correctly when using only our uncalibrated measurements, 70.5% when using only clinical variables, and 72.2% when combining both (performance results in terms of sensitivity, specificity, and area under the ROC are also shown in the table). Again these results are similar to those reported by Lee et al. (also shown in Table 5): an accuracy of 71.1% for uncalibrated measures, 76.1% for clinical variables (age, BMI, and witnessed apneas), and 79.4% for clinical (witnessed apneas; modified Mallampati class) and calibrated measures. Despite this similar trend, our results show lower OSA discrimination for all performance metrics (specially when comparing accuracy results for craniofacial features + clinical variables: 72.2% versus 79.4%). These differences might not be statistically significant considering the confidence intervals when developing the classification models; for example, Lee et al. [5] indicate that the assessment of the accuracy during their cross-validation process exhibited a range of 61% to 76%, which includes the accuracy found in our results. Nevertheless, it could be worth analyzing other reasons for this loss of accuracy besides the obvious ones that derive from our use of a prediction technique (SVR) for classification and less controlled uncalibrated measurements. There may be some differences in the populations under study, though not in terms of OSA prevalence, as there are similar values in both studies. However, the population explored by Lee et al. [5] includes both males (76.1%) and females (23.9%) while in our case only male individuals are studied. Lee et al. [5] do not provide information on the male/female balance in OSA and non-OSA groups, and the significantly lower prevalence of OSA in women compared to men [42] together with the differences between female and male craniofacial OSA risk factors [43] may introduce some bias in the performance results. As an illustration of this, in [32] we have shown how, due to the notable differences between female and male voices [44], when using speech acoustic features for OSA assessment over OSA and non-OS populations with imbalanced female/male proportions [20], clearly overoptimistic discrimination results are obtained.

It is also interesting to remark that in [5] only two clinical variables, witnessed apneas (i.e., the bed partner reporting witnessed apneas in a questionnaire) and modified Mallampati class (a visual classification of the anatomy of the oral cavity used to identify OSA patients) [45], were found to improve the classification accuracy when combining facial and clinical variables. As authors point out, this suggests that calibrated measurements are more important predictors than age and BMI (and highly correlated with them), while witnessed apneas and Mallampati score provide complementary information. Unfortunately, at present we do not have information on witnessed apneas and Mallampati score for all the individuals in our database. Nevertheless, according to our results, both OSA detection and AHI estimation (see Tables 2–5) show improvements when uncalibrated measures are combined with age, BMI, and cervical perimeter. We may thus hypothesize that the possible loss in OSA detection performance due to our less precise uncalibrated facial measurements can be compensated when adding information from easily available clinical variables (age, BMI, and cervical perimeter). Additionally, based also on Lee at al. results, we should expect further improvements if we were able to add information on witnessed apneas and Mallampati score. We believe that, looking for a simple application on a mobile device, information for witnessed apneas can be easily obtained from the patient itself. For the Mallampati score, though its use for OSA detection is controversial [46], we are currently working on its automatic estimation through image processing techniques on photography of patient's oropharynx.

Another relevant point to discuss, already stated in [5], is that facial measurements related to size (e.g., face width) seem to be better OSA predictors than facial shapes. This will guide our future research towards two complementary lines. On the one side, because we were looking for a usable scenario, we did not include any calibration procedure that could have allowed us to convert pixel measurements to metric dimensions. Our future research will explore users' acceptance to follow simple procedures, for example, sticking a calibration marker in their faces (as a circular nylon washer [4]), and we will evaluate the expected OSA detection improvement from the use of calibrated measurements (i.e., sizes) in our application scenario. On the other side, we believe that beyond simple measurements from facial landmarks the extraction of richer information related to facial shapes and textures using more powerful image processing techniques [47–49] can provide new insights into the OSA craniofacial phenotype and even characterize the sleepy facial appearance [50].

We acknowledge several limitations in our work that should be addressed in future research. Predictive results presented in this paper are limited to a particular sleep clinical population; consequently other clinical and general community populations should be studied to assess the clinical utility of our models. We have only studied male Caucasian subjects, and both sex and ethnicity can be relevant factors mainly related to facial measurements and speech features. Our database currently includes an important number of females, so the extension of this study on female individuals could be especially interesting as apnea disease is still not well understood in women.

The weak correlation observed between speech and OSA can be limited by the modelling approach we have followed (MFCC and i-vectors), and different results could be obtained using other acoustic representations or modelling approaches. For example, different techniques to compensate unwanted sources of variability in the i-vector subspace, as those proposed in [25, 51], could be considered.

We also have to acknowledge the limitation of recording speech at night when patients go to the sleep unit. Other acoustic differences could be expected at different times of the day, for example, soon after waking in the morning voice could reflect laryngeal trauma from overnight snoring. In fact, recent research has already pointed at other possible effects on patient voices that can help in OSA detection as analyzing speech recorded from a supine or stretched position [20, 23]. Even promising results have been reported from the acoustic analysis of nose and mouth breath sounds recorded in supine and upright positions [52, 53]. Our results are also limited to speech from Spanish speakers, so comparisons with other languages will require a careful analysis of language-dependent acoustic traits in OSA voices.

Finally, to evaluate the negative impact of different factors generally present in real scenarios, future studies will need to examine the effect of using different mobile devices with different cameras and microphones, testing the effect of different illumination conditions, as well as variable camera orientations and distances.

## 5. Conclusions

Frontal and profile images and voice recordings collected from a clinical population of 285 males were used to estimate the AHI using image and speech processing techniques. State-of-the-art speaker's voice characterization based on i-vectors only achieved a very weak performance for AHI estimation. Better prediction results were attained when using three uncalibrated photographic measurements calculated after detecting facial landmarks. Our experimental results show that OSA prediction can be improved when combining both clinical and facial measurements. The analysis of these results, contrasted to relevant previous research, points at several ways of improvement that can make OSA detection possible in practical scenarios using mobile devices and automatic speech and image processing.

## Figures and Tables

**Table 1:** Descriptive statistics on the 285 male subjects.

Clinical variables	Mean	SD^*∗*^	Range
AHI	21.7	17.4	0.0–84.4
Weight (kg)	92.5	16.9	61.0–162.0
Height (cm)	175.7	7.1	157.0–197.0
BMI (kg/m^2^)	30.0	5.0	20.0–52.3
Age (years)	48.4	12.0	21.0–85.0
Cervical Perimeter (cm)	42.3	3.1	34.0–52.0

AHI: Apnea-Hypopnea Index; BMI: Body Mass Index.

^*∗*^SD: standard deviation.

**Table 2:** AHI estimation using clinical variables.

Feature	MAE	Correlation coefficient (CC)
Clinical variables	12.32	0.40

The correlation coefficients (CC) are significant beyond the 0.001 level of confidence.

**Table 3:** AHI estimation using craniofacial measures.

Feature	MAE	Correlation coefficient (CC)
Uncalibrated craniofacial features	12.56	0.37
Uncalibrated craniofacial features + clinical variables	11.97	0.45

The correlation coefficients (CC) are significant beyond the 0.001 level of confidence.

**Table 4:** AHI estimation using i-vectors.

Feature	MAE						Correlation coefficient (CC)					
	i-vector dimension						i-vector dimension					
	400	300	200	100	50	30	400	300	200	100	50	30
i-vector	13.79	13.86	14.20	14.05	13.79	14.05	0.08	0.09	0.05	0.09	0.13	0.08
i-vector + clinical variables	12.80	12.43	12.48	12.63	12.55	12.68	0.33	0.38	0.38	0.36	0.38	0.37

The correlation coefficients (CC) are significant beyond the 0.05 level of confidence.

**Table 5:** Classification results of prediction of OSA using AHI estimated.

Feature	Accuracy	Sensitivity	Specificity	ROC AUC
Clinical variables	70.5%	72.6%	57.5%	0.72
Clinical variables, Lee et al. [5]	**76.1%**	**86.0%**	**59.1%**	**0.78**
Uncalibrated craniofacial features	70.8%	71.8%	62.1%	0.67
Uncalibrated craniofacial features Lee et al. [5]	**71.1%**	**80.7%**	**54.5%**	**0.80**
Uncalibrated craniofacial features + clinical variables	72.2%	73.3%	64.8%	0.73
Calibrated craniofacial features + clinical variables, Lee et al. [5]	**79.4%**	**85.1%**	**69.7%**	**0.87**
